# GWAS follow-up study of esophageal squamous cell carcinoma identifies potential genetic loci associated with family history of upper gastrointestinal cancer

**DOI:** 10.1038/s41598-017-04822-2

**Published:** 2017-07-05

**Authors:** Xin Song, Wen-Qing Li, Nan Hu, Xue Ke Zhao, Zhaoming Wang, Paula L. Hyland, Tao Jiang, Guo Qiang Kong, Hua Su, Chaoyu Wang, Lemin Wang, Li Sun, Zong Min Fan, Hui Meng, Tang Juan Zhang, Ling Fen Ji, Shou Jia Hu, Wei Li Han, Min Jie Wu, Peng Yuan Zheng, Shuang Lv, Xue Min Li, Fu You Zhou, Laurie Burdett, Ti Ding, You-Lin Qiao, Jin-Hu Fan, Xiao-You Han, Carol Giffen, Margaret A. Tucker, Sanford M. Dawsey, Neal D. Freedman, Stephen J. Chanock, Christian C. Abnet, Philip R. Taylor, Li-Dong Wang, Alisa M. Goldstein

**Affiliations:** 1 Henan Key Laboratory for Esophageal Cancer Research, The First Affiliated Hospital of Zhengzhou University, 40 Daxue Road, Zhengzhou, Henan 450052 P.R. China; 2 Division of Cancer Epidemiology and Genetics, National Cancer Institute, National Institute of Health, Bethesda, MD USA; 3 Department of Dermatology, Warren Alpert Medical School, Brown University, Providence, RI USA; 4 Department of Epidemiology, School of Public Health, Brown University, Providence, RI USA; 5 Cancer Genomics Research Laboratory, Leidos Biomedical Research, Frederick National Laboratory for Cancer Research, Frederick, MD USA; 6 Department of Computational Biology, St. Jude Children’s Research Hospital, Memphis, TN USA; 7 Department of Gastroenterology, The Fifth Affiliated Hospital of Zhengzhou University, Zhengzhou, Henan province 450052 China; 8 Department of Pathology, Cixian Hospital, Cixian, Hebei 056500 P.R. China; 9 Department of Thoracic Surgery, Anyang Tumor Hospital, Anyang, Henan 455000 P.R. China; 10 Shanxi Cancer Hospital, Taiyuan, Shanxi P.R. China; 11 Department of Epidemiology, Cancer Institute (Hospital), Chinese Academy of Medical Sciences, Beijing, P.R. China; 12 Information Management Services, Inc., Silver Spring, MD USA

## Abstract

Based on our initial genome-wide association study (GWAS) on esophageal squamous cell carcinoma (ESCC) in Han Chinese, we conducted a follow-up study to examine the single nucleotide polymorphisms (SNPs) associated with family history (FH) of upper gastrointestinal cancer (UGI) cancer in cases with ESCC. We evaluated the association between SNPs and FH of UGI cancer among ESCC cases in a stage-1 case-only analysis of the National Cancer Institute (NCI, 541 cases with FH and 1399 without FH) and Henan GWAS (493 cases with FH and 869 without FH) data (discovery phase). The top SNPs (or their surrogates) from discovery were advanced to a stage-2 evaluation in additional Henan subjects (2801 cases with FH and 3136 without FH, replication phase). A total of 19 SNPs were associated with FH of UGI cancer in ESCC cases with *P* < 10^−5^ in the stage-1 meta-analysis of NCI and Henan GWAS data. In stage-2, the association for rs79747906 (located at 18p11.31, *P* = 5.79 × 10^−6^ in discovery) was replicated (*P* = 0.006), with a pooled-OR of 1.59 (95%CI: 1.11-2.28). We identified potential genetic variants associated with FH of UGI cancer. Our findings may provide important insights into new low-penetrance susceptibility regions involved in the susceptibility of families with multiple UGI cancer cases.

## Introduction

Esophageal cancer (EC) represents the sixth most frequent cause of cancer-related deaths worldwide^1, 2^. Over half of all EC-related deaths occur in China where esophageal squamous cell carcinoma (ESCC) is the predominant histologic subtype, particularly in high-risk populations^3, 4^. Both genetic and environmental risk factors are believed to play roles in the development of ESCC. In Western populations, smoking and heavy alcohol intake are established dominant risk factors for ESCC^5^. However, smoking and alcohol are not major contributing factors in the high-risk populations in north central China^6, 7^, where causes underlying the carcinogenesis of ESCC remain poorly defined.

We and others have conducted several genome-wide association studies (GWAS) and identified a number of genetic loci linked to risk of ESCC^8–15^, but these loci account for only a small fraction of the genetic susceptibility for ESCC risk. Previous studies in the high-risk populations in north central China have demonstrated consistent associations between family history (FH) of cancer, particularly that of upper gastrointestinal (UGI) cancer, and risk of ESCC^16–18^. This strong tendency toward familial aggregation suggests the potential usefulness of looking at genetic predisposition for UGI cancer by examining family-history-related genetic loci. Based on our initial GWAS for ESCC in high-risk populations of Han Chinese ethnicity^9, 10^, we conducted additional analyses of the association of single nucleotide polymorphisms (SNPs) with FH of UGI cancer in ESCC cases. First, SNPs consistently associated with FH of UGI cancer in a case-only analysis of two GWAS were identified (discovery stage)^9, 10^. Second, top SNPs associated with FH of UGI cancer in the GWAS (or their surrogate SNPs) were evaluated in a second stage replication in additional cases.

## Results

### Meta-analysis of GWAS for the discovery stage

A total of 19 SNPs were associated with FH of UGI cancer in ESCC cases with a *P* < 0.05 in both the National Cancer Institute (NCI) and Henan GWAS data in the discovery stage (Supplementary Table 1) and a combined *P* < 10^−5^ in the meta-analysis (Tables 1 and 2). None reached genome-wide significance. The smallest *P* value was observed for rs140792366 (*P* = 7.65 × 10^−7^), which is in the gene *GRIK4* (located at 11q23.3).

**Table 1:** The association between rs79747906 (T-C) and family history in ESCC cases.

	No. of cases with FH	No. of cases without FH	MAF for cases with FH	MAF for cases without FH	OR^**a**^ (95% CI)	*P* ^**b**^
**GWAS discovery**						
NCI study^b,c^	541	1399	0.068	0.051	1.70 (1.15–2.51)	0.007
Henan study^b^	493	869	0.081	0.053	2.16 (1.44–3.23)	0.0002
Meta-analysis^d^	1034	2268	0.074	0.052	1.91 (1.44–2.52)	5.79 × 10^−6^
**Henan replication**						
Model 1^e^	2801	3136	0.076	0.062	1.23 (1.06–1.42)	0.006
Model 2^f^	1937	3136	0.076	0.062	1.24 (1.06–1.46)	0.008

FH: family history; MAF: minor allele frequency; odds ratio: OR.

^a^The *P*-values and ORs per one effect allele (the minor allele C) were calculated from unconditional logistic regression models using genotype-trend tests adjusted for age, sex and sub-study (for the analysis of NCI study, which includes NCI Shanxi and NIT).

^b^ESCC cases with FH of UGI cancer (FH+) were defined as those having one or more relative(s) diagnosed with UGI cancer within three generations for NCI/Shanxi study and Henan study.

^c^For the smaller NCI study (NIT), FH was limited to those having one or more relative(s) diagnosed with UGI cancer within first-degree relatives.

^d^Calculated using the fixed-effects model as no heterogeneity was found between the two studies (*P* = 0.41).

^e^ESCC cases with family history were defined as those that have one or more relative(s) within three generations diagnosed with UGI cancer.

^f^ESCC cases with family history were defined as those that have one or more relative(s) within first-degree diagnosed with UGI cancer.

**Table 2:** Top SNPs in the NCI and Henan GWAS associated with family history of UGI cancer in ESCC cases^a^.

Gene	Chr.	Discovery phase (Meta-analysis of NCI and Henan GWAS)							Replication phase (Henan)						
		SNP (major, minor)	n1	n2	A1	A2	OR^b^	*P* ^b^	SNP (major, minor)	n1	n2	A1	A2	OR^b^	*P* ^b^
*GRIK4*	11q23.3	rs140792366 (C, G)	1032	2266	0.027	0.015	4.23	7.65 × 10^-7^	rs140792366 (C, G)	2801	3135	0.006	0.005	N/A^f^	N/A^f^
*BC047542*	2q37.1	rs117453803 (A, T)	1032	2266	0.075	0.052	2.07	9.74 × 10^-7^	rs73997003 (G, A)^c^	2801	3136	0.074	0.067	1.10	0.19
	17p13.2	rs187481103 (A, C)	1032	2267	0.024	0.013	4.08	1.27 × 10^−6^	rs184911713 (G)^d^	2801	3135	0	0	N/A^f^	N/A^f^
*COLA1L,COL21A1*	6p12.1	rs9357885 (A, T)	1032	2266	0.179	0.139	1.49	1.48 × 10^−6^	rs6459122 (C, T)^c^	2799	3135	0.091	0.094	0.97	0.65
	2p24.1	rs2049728 (T, C)	1032	2268	0.255	0.310	0.76	3.81 × 10^−6^	rs2049728 (T, C)	2799	3135	0.289	0.296	0.97	0.38
	2q32.1	rs57921607 (A, C)	1032	2266	0.163	0.131	1.60	4.07 × 10^−6^	N/A^e^	N/A^e^	N/A^e^	N/A^e^	N/A^e^	N/A^e^	N/A^e^
*CYP19A1*	15q21.2	rs186503151 (C, T)	1033	2268	0.026	0.014	3.63	4.14 × 10^−6^	rs141703242 (C)^d^	2801	3136	0	0	N/A^f^	N/A^f^
	7q35	rs2372415 (C, G)	1032	2266	0.282	0.230	1.35	4.28 × 10^−6^	rs2372415 (C, G)	2799	3135	0.255	0.258	0.99	0.79
	3p22.3	rs12631160 (T, C)	1033	2267	0.047	0.027	2.10	5.35 × 10^−6^	rs12631160 (T, C)	2799	3135	0.047	0.047	1.02	0.85
*DLGAP1*	18p11.31	rs79747906 (T, C)	1032	2266	0.074	0.052	1.91	5.79 × 10^−6^	rs79747906 (T, C)	2801	3136	0.076	0.062	**1.23**	**0.006**
*STK31*	7p15.3	rs1549391 (C, T)	1033	2267	0.040	0.069	0.59	6.32 × 10^−6^	rs1549391 (C, T)	2801	3136	0.052	0.051	1.05	0.59
*NXPH1*, *hCG_2009575*	7p21.3	rs2285970 (T, C)	1032	2266	0.037	0.019	2.43	6.41 × 10^−6^	rs2285970 (T, C)	2799	3135	0.030	0.036	0.83	0.08
	3p22.3	rs1073209 (T, C)	1033	2268	0.095	0.067	1.61	6.52 × 10^−6^	rs4955227 (T, G)^c^	2801	3136	0.074	0.071	1.04	0.54
*C12orf5*, *FGF23*	12p13.32	rs1046165 (T, C)	1034	2268	0.529	0.465	1.28	7.14 × 10^−6^	rs1046165 (T, C)	2801	3136	0.484	0.479	1.02	0.60
	20q13.31	rs59635584 (G, T)	1032	2266	0.035	0.020	2.54	7.37 × 10^−6^	rs59635584 (G, T)	2801	3136	0.019	0.020	0.97	0.80
*ATP1B2*, *TP53*, *p53*	17p13.1	rs1050533 (T, C)	1032	2266	0.508	0.457	1.30	8.56 × 10^−6^	rs1050541 (T, G)^c^	2801	3135	0.424	0.410	1.05	0.19
*UCA1*	19p13.12	rs12461816 (C, T)	1032	2266	0.218	0.176	1.39	8.91 × 10^-6^	rs12461816 (C, T)	2801	3136	0.209	0.202	1.04	0.37
*COLA1L*, *COL21A1*	6p12.1	rs2745751 (A, C)	1033	2267	0.112	0.080	1.54	8.96 × 10^-6^	rs1883703 (A, T)^c^	2799	3135	0.080	0.082	0.98	0.72
*MPDU1*, *FXR2, SHBG*	17p13.1	rs58614441 (T, C)	1032	2266	0.186	0.148	1.43	9.11 × 10^-6^	rs34416693 (G, A)^c^	2800	3135	0.237	0.230	1.03	0.57

^a^n1 is the number of cases with family history while n2 is the number of cases without family history. A1 is the allele frequency of the minor allele (effect allele) in cases with family history and A2 is the allele frequency of the minor allele (effect allele) in cases without family history. SNPs are ordered based on the increasing *P*-values in the discovery phase.

^b^The *P*-values and ORs for the SNP (per one minor allele) were calculated from unconditional logistic regression models using genotype-trend tests adjusted for age, sex and sub-study (for the analysis of NCI study, which includes NCI Shanxi and NIT).

^c^Surrogate SNPs were selected to replace the original SNP from the meta-analysis of the NCI and Henan GWAS. The surrogates were located within 200 kb on either side of each targeted SNP and were selected based on the r^2^ with the targeted SNP in the genotype data from 1000 Genomes project JPT + CHB population. The linkage r^2^ was 1.00 for rs73997003 with rs117453803, 0.62 for rs6459122 with rs9357885, 0.93 for rs4955227 with rs1073209, 0.58 for rs1050541 with rs1050533, 0.94 for rs1883703 with rs2745751, and 0.59 for rs34416693 with rs58614441.

^d^For rs187481103 and rs186503151, we were unable to find good surrogates that could survive the assay design. Given the monoallelic nature of the tested surrogates (rs184911713 and rs141703242) in our study population, we were essentially unable to test/replicate the findings for those two SNPs.

^e^This SNP (rs57921607) and its surrogate(s) failed in the follow-up study.

^f^ORs and P values not calculated because of the extremely rare minor allele.

### SNP Replication analysis in Henan Sample

Of these 19 SNPs, rs57921607 and its surrogate failed design and were dropped from further consideration. Ten original and 8 surrogate SNPs were genotyped in the replication stage (Table 2 and Supplementary Table 1). Among the 18 SNPs tested in replication, we did not see an alternative (minor) allele for rs141703242 (surrogate of rs186503151) or rs184911713 (surrogate of rs187481103). Further, we found very few minor alleles for rs140792366. Association analyses were, therefore, limited to the remaining 15 SNPs. The candidate SNP with strongest associations with FH in the discovery (rs140792366) had much lower MAF in the stage 2 and was not replicated. Rs79747906 in *DLGAP1* (located at 18p11.31, odds ratio [OR] = 1.91 and *P* = 5.79 × 10^−6^ in the meta-analysis of discovery set) had the smallest p-value in the replication analysis with FH defined for relatives in three generations and was significant at a nominal significance level (OR = 1.23, *P* = 0.006) (Tables 1 and 2). Based on the random-effect model (*P* for heterogeneity = 0.02), the pooled OR (95% CI) for the discovery and replication set was 1.59 (1.11–2.28) (*P* = 0.01). The association for rs79747906 was also evident in the secondary model of replication analysis, for first degree relatives only (OR = 1.24, *P* = 0.008) (Table 1 and Supplementary Table 2). The pooled OR (95% CI) for the second replication model and the discovery set was 1.59 (1.12–2.26) (*P* = 0.009).

We also found a suggestive association for rs12461816 (located at 19p13.12) and FH in the secondary replication model (OR = 1.39, *P* = 8.91 × 10^−6^ in the discovery set, and OR = 1.09, *P* = 0.08 in replication; the pooled OR = 1.27, *P* = 0.03) (Table 2 and Supplementary Table 2). However, the primary analysis did not support a significant association for this SNP (OR = 1.04 and *P* = 0.37) (Table 2).

### In silico and cis-eQTL functional annotation

We annotated the two SNPs (rs79747906 and rs12461816) that showed suggestive association with FH in the replication. The functional analysis revealed that rs79747906 C allele may have a weak CTCF binding function. SNP rs12461816 has a potential weak polycomb-repressed state of transcription in stomach smooth muscle and the T allele of rs12461816 abolishes a methylated CpG in normal esophagus and gastric tissues (Table 3 and Supplementary Figures 1 and 2).

**Table 3:** Functional annotation of rs79747906 and rs12461816.

SNP	rs79747906 (risk allele C, 18p11.31)	rs12461816 (risk allele T, 19p13.12)
Gene Annotation	*DLGAP1*, intergenic	*UCA1*, intergenic
Chromatin Regulatory States^a^	None	Stomach Smooth Muscle (ReprPC_W)
DNaseI Site^b^	No	No
Alters CpG^c^	No	Yes, methylated CpG in normal esophagus and gastric tissues
Protein(s) bound^d^	Weak CTCF binding	None
DNA Motif(s) altered^e^	CDP, Nanog, PAX4 and SMAD2	MSX-1
eQTL (risk allele association, *P*) in esophagus^f^	No significant association^g^	*AC004791.2* (β = 0.60, *P* = 6.00 × 10^−7^) Muscularis *CYP4F24P* (β = 0.66, *P* = 4.50 × 10^−4^) GEJ *AC004791.2* (β = 0.37, *P* = 7.30 × 10^−4^) Mucosa *CYP4F11* (β = −0.37, *P* = 8.50 × 10^−4^) Mucosa
eQTL (risk allele association, *P*) in stomach^f^	No significant association^g^	*AC004791.2* (β = 0.67, *P* = 9.50 × 10^−7^)
eQTL (risk allele association, *P*) in blood^f^	No significant association^g^	*AC004791.2* (β = 0.38, *P* = 2.5 × 10^−5^)
Number of eQTL gene tests (significance *P*)	17 tests (*P* < 2.94 × 10^−3^)	57 tests (*P* < 8.77 × 10^−4^)

^a^Chromatin State Segmentation using a Hidden Markov Model (ChromHMM) by Auxillary Core Marks + K27Ac in adipose derived mesenchymal stem cells (ASC), adipose nuclei (AN), blood cells (CD19, CD8 and CD4), stomach mucosa (SM), stomach smooth muscle (SMM), gastric and esophagus tissues issues from Roadmap (http://www.roadmapepigenomics.org/). Repressed histone methylation (Re), Repressed polycomb weak (ReprPC_W), Repressed polycomb(ReprPC), Heterochromatin (H), Transcription (T), Quiescent (Q), and Enhancer (E).

^b^DNaseI Hypersensitivity sites were assigned by DNase I hypersensitive assay in 125 cell types in ENCODE (http://genome.ucsc.edu/ENCODE/) and in NIH Roadmap data.

^c^DNA CpG altered in esophagus and stomach tissues from NIH Roadmap data.

^d^Transcription binding sites and binding motif defined by chromatin immunoprecipitation sequencing for 161 factors were obtained from ENCODE and HaploReg v4.

^e^DNA binding motifs for transcription factors (TF) and proteins obtained from HaploReg v4. DNA motifs were assigned to a factor group based on TF ChIP experiments and known DNA motifs as described by Keheradpour P and Kellis M^25^.

^f^Expression quantitative trait loci (eQTL) analysis were conducted in normal esophageal tissues: esophageal mucosa[Mucosa]; esophageal muscularis [Muscularis]; and gastroesophageal junction [GJE], normal stomach mucosa, and the whole blood. We examined coding and non-coding genes *in cis* or located within a 1MB of the signal and known to be expressed at the mRNA in the target tissue. Results were obtained from the Genotype-Tissue Expression (GTEx) Project (http://www.gtexportal.org/home/). *P-*values were calculated based on linear regression between log and quantile normalized RNA-seq expression values and imputation-based genotype with 3 genotyping principal components, 15 peer factors, and gender as covariates.

^g^No significant association after considering multiple comparisons for number of tests. Suggestive associations with P < 0.05 are shown in SI Table 3.

In the expression quantitative trait loci (eQTL) analyses, rs12461816 T allele was significantly associated with increased *cis* expression of *AC004791.2* in normal esophageal muscularis (*P* = 6.00 × 10^−7^) and mucosa (*P* = 7.30 × 10^−4^), normal stomach mucosa *(P* = 9.50 × 10^−7^), and whole blood (*P* = 2.5 × 10^−5^). It was also associated with increased expression of *CYP4F24P* (*P* = 4.50 × 10^−4^) in the gastroesophageal junction, decreased expression of *CYP4F11* (*P* = 8.50 × 10^−4^) in esophageal mucosa, and showed nominal associations with several other genes (Table 3 and Supplementary Table 4). For rs79747906, the C allele was suggestively associated with altered expression of several genes such as *LPIN2*, although none of these associations were significant after adjustment for multiple comparisons (Supplementary Tables 3 and 4).

## Discussion

We conducted analyses based on our initial GWAS for ESCC, examining the SNPs associated with FH of UGI cancer in Han Chinese. We found 19 SNPs consistently associated with FH of UGI cancer in two GWAS combined. Of these, rs79747906 (18p11.31) was replicated in analyses of additional cases. To our knowledge, no previous studies have reported this SNP as associated with FH of UGI cancer or UGI cancer risk.

Rs79747906 is located in the intergenic region close to *DLGAP1*, SNPs of which have been associated with obsessive-compulsive disorder^19^. Based on Roadmap Epigenomics data rs79747906 does not appear to map to a regulatory region in normal UGI tissues or blood, but the C allele, which was positively associated with FH, overlapped with a weak CTCF binding function in ENCODE cells including Human Esophageal Epithelial Cells (HEEpiC) (Table 3 and SI Figure 1). However, we did not observe potential regulatory function for any SNPs in LD with rs79747906 (r^2^ ≥ 0.40, http://analysistools.nci.nih.gov/LDlink/) in normal esophageal tissue (data not shown). We also found that the rs79747906 C allele may alter several DNA binding motifs of homeobox transcriptional factors and proteins, including CDP, Nanog, PAX4, and SMAD2 (ENCODE and HaploRegv4) and scores high as a regulatory SNP for embryonic stem and progenitor cells of other tissues (RegulomeDB and HaploRegv4). Collectively, these findings suggest that the genetic region containing rs79747906 has the potential to change chromatin architecture in esophageal epithelia and/or in esophageal stem cells via protein binding. Our eQTL analysis of rs79747906 C allele showed only suggestive and tissue-specific evidence of a potential *cis*-eQTL effect on transcription, such as increased expression of *LPIN2* (more distal protein coding gene) in blood and *RP13-270P17.3* (a long intergenic non-coding RNA [LincRNA] gene) in stomach mucosa. It is worth noting that metabolic changes have been reported for UGI cancer patients previously^20, 21^. LPIN2 protein is known for its role in metabolism, with *LPIN2* SNPs associated with metabolic traits^22^, which may be important for UGI cancer. Our study provides evidence for future examination of *LPIN2* and *RP13-270P17.3* and risk of UGI cancer.

The T allele of rs12461816 (19p13.12) was associated with FH and can abolish a methylated CpG in normal esophagus and stomach tissues. In keeping with a methylated and condensed DNA status, rs12461816 is also located to a potential polycomb-repressed DNA region in stomach smooth muscle, suggesting a possible transcriptional repression function for this region regulated by epigenetics. Notably, rs12461816 T allele was strongly associated with increased expression of *AC004791.2*. The expression of multiple other genes with important functions may be altered by rs12461816 variant too, such as *CYP4F11*, which encodes a cytochrome P450 enzyme (and is important for arachidonic acid or fatty acid metabolism).

UGI cancer is a major public health concern worldwide, among the most frequent causes of cancer-related deaths^1, 2^, highlighting the urgent need to elucidate the mechanisms underlying carcinogenesis. We identified potential genetic variants associated with FH of UGI cancer in ESCC cases. Our findings may provide important insights into new low-penetrance susceptibility regions not only for ESCC but possibly for UGI cancer overall, contributing to the understanding of ESCC and UGI cancer pathogenesis. Further investigation of the underlying genetic susceptibility may reveal new pathways predisposing to UGI cancer and potentially identify new therapeutic targets for drug discovery, aiding in the prevention and management of these common tumors.

## Methods

### Study population

#### Discovery stage sample

The discovery stage was based on the NCI GWAS and Henan GWAS. Participants for the NCI GWAS were drawn from the Shanxi UGI Cancer Genetics Project with participants residing in the western Taihang Mountain area; and the Nutrition Intervention Trials (NITs), with participants from the southern Taihang Mountain area. The Shanxi study was conducted between 1997 and 2007 and included case-control and case-only study components. Newly-diagnosed, histologically-confirmed ESCC and gastric cancer cases were identified and blood samples collected at enrollment for all cases^7^. The NITs were initiated in Linxian in 1985 and tested the effect of multiple vitamin and mineral combinations taken for up to six years on the incidence and mortality of EC and gastric cardia cancer^4^. Following a blood survey conducted in 1999–2000, all newly-diagnosed, histologically-confirmed ESCC cases documented during the follow-up through December 31, 2007, were included in the current analysis. Both the Shanxi and NIT studies were approved by their respective Institutional Review Boards and written informed consent was obtained from all subjects prior to participation. The NCI Special Studies Institutional Review Board approved both the Shanxi and NIT studies as well as the overall GWAS.

Participants for the Henan GWAS were collected from an ongoing hospital-based ESCC case-control study from northern China. The cases for the current study were restricted to the ‘genetically matched’ subset pool that was obtained from within Henan province. The study was approved by each institutional and hospital ethical committee and conducted according to the principles of the Declaration of Helsinki.

Our study was restricted to ESCC cases only as all traditional controls in the Henan GWAS were selected originally to have a negative family history, thus precluding an examination of FH differences using controls.

#### Replication stage sample

Replication of the top SNPs in the discovery stage was based on additional ESCC cases from Henan. Participants for the replication stage were part of the same ongoing hospital-based ESCC case-control study as for the Henan GWAS, but case ascertainment occurred subsequent to the Henan GWAS study. Similarly, the study population for the replication stage was restricted to ESCC cases only.

#### Family history of UGI cancer

For all studies from the discovery stage and replication stage, information on the diagnosis of UGI cancer, including age at diagnosis, gender, and tumor type, within family members for each study participant was collected through questionnaire by interview.

In the discovery stage, FH was defined according to available information to maximize the number of subjects, as available data on FH varied slightly for the three studies in the discovery stage. For NCI/Shanxi study and Henan study, ESCC cases with FH of UGI cancer (FH+) were primarily defined as those having one or more relative(s) diagnosed with UGI cancer within three generations. ESCC cases without FH of UGI cancer (FH−) were defined as those who did not have any relatives diagnosed with esophageal or gastric cancer within three generations. For the smaller NCI study (NIT), definition of FH was limited to first-degree relatives, with FH+ defined as one or more first-degree relatives diagnosed with UGI cancer. In sum, the discovery of the SNPs associated with FH of UGI cancer in cases of ESCC was based on a total of 541 cases with FH and 1399 without FH from the two NCI GWAS, including 343 cases with FH and 1076 without FH in NCI/Shanxi study, and, 198 cases with FH and 323 without FH in NIT, and 493 cases with FH and 869 without FH from the Henan GWAS.

For the replication phase sample, we considered two different definitions of a positive FH of UGI cancer: For the primary analysis, we used the same FH definition used for the majority of the discovery stage (NCI/Shanxi and Henan), in which we defined FH+ as having one or more relatives within three generations diagnosed with UGI cancer. Based on this definition, we have 2801 cases with FH and 3136 without FH. We also examined a more conservative definition of FH (limited to having one or more relatives with UGI cancer within first-degree relatives) to make sure that results did not differ based on FH definition; this secondary analysis included 1937 cases with FH and 3136 without FH.

### Genotyping and quality control

#### GWAS data for the discovery stage

The details of the analytic preprocessing for the NCI GWAS and Henan GWAS were described previously^9, 10^. Additional quality control procedures were implemented in a joint analysis that included these two GWAS^13, 15^. Specifically, SNPs with a call rate < 95%, a Hardy-Weinberg proportion test P < 0.000001 or a MAF < 1% were excluded before the subsequent imputation analysis that was conducted separately for each of the two GWAS scans. Details of the imputation analysis have been previously published^15^.

#### SNP Replication in Henan Sample

A total of 19 top SNPs associated with FH of UGI cancer in the discovery stage (*P* < 0.05 in each individual GWAS and < 10^−5^ in the meta-analysis) were further examined in the 5937 additional Henan subjects. We tested the original SNPs (10 of 19) or their surrogates (8 of 19) if the original SNP failed assay design. The surrogate SNPs were selected by searching within 200 kb on either side of each targeted SNP. We used the genotype data from 1000 Genomes project JPT + CHB population to estimate the pair-wise LD and selected the three best LD surrogates based on r^2^ values for each targeted SNP. If the best one (that is, the SNP with the highest r^2^ with the targeted SNP) failed the assay design, we nominated the second SNP. If the second one also failed the assay design, we then nominated the third SNP as the surrogate. The r^2^ between the surrogate SNP and the original SNP is included in the footnotes of Table 2 and Supplementary Table 2. One SNP was dropped because both the original SNP and its only available surrogate failed assay design.

The genotyping of the 18 replication SNPs was performed as follows: a segment of DNA which surrounded the SNPs (100 bp) was amplified through PCR by using HotStarTaq (Qiagen). After purification by shrimp alkaline phosphatase and exonuclease I (Epicentre), the PCR products were tested by a primer extension assay by using the SNaPshot Multiplex kit (ABI). An ABI 3130xl capillary electrophoresis DNA instrument with Gene Mapper 4.0 software (Applied Biosystems, Foster City, CA) was used to analyze the resulting primer extension products.

### Statistical analysis

#### Meta-analysis of GWAS for the discovery stage

Details of the statistical analysis methods for the NCI GWAS and Henan GWAS were included in the primary reports^9, 10^. We conducted meta-analyses to combine the β-estimates and standard errors from each GWAS. We tested the between-study heterogeneity and estimated the overall association from the fixed-effects model (weighted proportionately to the inverse of the study-specific variance). We identified eigenvectors for each GWAS and included the significant eigenvectors (*P* < 0.05) to control for population stratification in each individual GWAS.

#### Replication analysis

For 18 SNPs (including 10 original SNPs and 8 surrogates), we examined the association between each SNP and FH by comparing FH+ ESCC cases to FH- cases. The *P*-values and ORs for the SNPs (per one minor allele) were calculated from unconditional logistic regression models using trend tests adjusted for age, and sex. The definition of FH+ in the primary and secondary models is detailed in the Study Population section. For SNPs with suggestive associations, the pooled ORs were calculated based on random effect models for meta-analysis, as significant heterogeneity was found between the discovery and the replication dataset.

#### In silico and cis-eQTL functional annotation

To explore whether SNPs associated with FH after replication stage might have potential regulatory functions, we used custom tracks on the UCSC Genome browser (http://genome.ucsc.edu) to screen Roadmap Epigenomics (http://www.roadmapepigenomics.org/) in esophageal and stomach tissues and blood, as well as ENCODE data for each implicated SNP region for evidence of regulatory relevance,^23, 24^ such as overlap with chromatin marks, CpG-site methylation, and transcription factor binding motifs^25^. We also used the online tools HaploRegv4 (http://www.broadinstitute.org/mammals/haploreg/haploreg.php) and RegulomeDB (http://regulome.stanford.edu) as complementary analyses to confirm the location of each SNP in relation to annotated protein-coding genes and/or non-coding RNA genes.

To identify SNPs associated with RNA expression, we used publically available data (Genotype-Tissue Expression Project [GTEx], http://www.gtexportal.org/home/) to perform eQTL analyses in relevant normal tissues, including normal esophageal mucosa (n = 241), esophageal muscularis (n = 218), gastroesophageal junction (n = 127), normal stomach mucosa (n = 170), and whole blood (n = 338). We assessed the impact of associated SNPs on coding and non-coding genes *in cis* or located within 1MB of the signal and known to be expressed at the mRNA in the target tissue from the GTEx Project. For each tissue type, we performed *cis*-eQTL analysis for each gene-SNP pair. Linear regression was conducted for the association between each SNP and log and quantile normalized RNA-sequencing expression values from each tissue, adjusting for three genotyping principal components, 15 peer factors, and sex (http://www.gtexportal.org/home/documentationPage). *P*-values were adjusted for multiple comparisons using Bonferroni correction (*P* = 0.05/total number of genes tested per risk locus).

## Electronic supplementary material

Supplementary information

Supplementary information

Supplementary information
