# Supplementary material for: GWAS follow-up study of esophageal squamous cell carcinoma identifies potential genetic loci associated with family history of upper gastrointestinal cancer
Source: Sci Rep. 2017 Jul 5;7:4642. doi: 10.1038/s41598-017-04822-2 (PMC5498542; doi:10.1038/s41598-017-04822-2)
Supplement: Supplementary file 1 — Supplementary information [file 41598_2017_4822_MOESM1_ESM.docx]

**GWAS follow-up study of esophageal squamous cell carcinoma identifies potential genetic loci associated with family history of upper gastrointestinal cancer**

Xin Song^1^**^*^,** Wen-Qing Li ^2,3,4 *^; Nan Hu ^2*^; Xue Ke Zhao^1*^; Zhaoming Wang^2,5,6^; Paula L. Hyland ^2^; Tao Jiang^1^ , Guo Qiang Kong^1^; Hua Su^2^; Chaoyu Wang^2^; Lemin Wang^2^; Li Sun^1^, Zong Min Fan^1^, Hui Meng^1^, Tang Juan Zhang^1^, Ling Fen Ji^1^, Shou Jia Hu ^1^, Wei Li Han ^1^, Min Jie Wu ^7^, Peng Yuan Zheng ^7^, Shuang Lv ^7^, Xue Min Li ^8^, Fu You Zhou ^9^; Laurie Burdett^2,5^; Ti Ding ^10^; You-Lin Qiao ^11^; Jin-Hu Fan^11^; Xiao-You Han^10^; Carol Giffen^12^; Margaret A. Tucker^2^; Sanford M. Dawsey ^2^; Neal D. Freedman ^2^; Stephen J*.* Chanock ^2^; Christian C. Abnet ^2^; Philip R. Taylor ^2^; Li-Dong Wang^1^**^**^,** Alisa M. Goldstein ^2**^

**Author affiliations:** ^1^ Henan Key Laboratory for Esophageal Cancer Research, The First Affiliated Hospital of Zhengzhou University, 40 Daxue Road, Zhengzhou, Henan, 450052, P. R. China; ^2^ Division of Cancer Epidemiology and Genetics, National Cancer Institute, National Institute of Health, Bethesda, MD, USA; ^3^ Department of Dermatology, Warren Alpert Medical School, Brown University, Providence, RI, USA; ^4^Department of Epidemiology, School of Public Health, Brown University, Providence, RI, USA; ^5^Cancer Genomics Research Laboratory, Leidos Biomedical Research, Frederick National Laboratory for Cancer Research, Frederick, MD, USA; ^6^Department of Computational Biology, St. Jude Children’s Research Hospital, Memphis, TN, USA; ^7^ Department of Gastroenterology, The Fifth Affiliated Hospital of Zhengzhou University, Zhengzhou, Henan province 450052, China; ^8^ Department of Pathology, Cixian Hospital, Cixian, Hebei 056500, P. R. China; ^9^Department of Thoracic Surgery, Anyang Tumor Hospital, Anyang, Henan 455000, P. R. China;^10^Shanxi Cancer Hospital, Taiyuan, Shanxi, P.R. China; ^11^Department of Epidemiology, Cancer Institute (Hospital), Chinese Academy of Medical Sciences, Beijing, P. R. China; ^12^Information Management Services, Inc, Silver Spring, MD, USA.

**Supplementary Figure 1.** Genome Browser (http://genome.ucsc.edu/) image of 18p11.31 genomic region containing rs79747906 on human assembly hg19 based on NIH Epigenomics Roadmap and ENCODE data. Coding & noncoding genes= Gencode Version 19 showing part of *DLGAP1* gene (and transcripts) and the noncoding RNA genes (*DLGAP1-AS3*, *RNU6-831P* and *DLGAP1-AS3*). CpG islands: Green bars. Regulatory regions= chromatin states from a hidden Markov Model [ChromHMM)] and core histone marks with H3K27 acetylation mark from normal blood cells [CD19 B cells and CD4 and CD8 T cells), esophagus, stomach mucosa (SM), gastric and stomach smooth muscle (SSM). Color key: Crimson, flanking promoter region; Hot Red, active promoter; Yellow: active enhancer; Dark gray, repressed polycomb region; Light gray, weak repressed polycomb region; and Light Blue, heterochromatin. SNPs= For clarity only the location of rs79747906 is shown from dbSNP142. Methylation= Bisulphite-sequencing of gastric and esophageal tissues (Roadmap). DNase clusters= Open chromatin (DNA) structure in 125 ENCODE cells lines. CTCF binding=Chip-seq analysis of CTCF binding to DNA. The region containing rs79747906 shows a weak interaction with CTCF binding in HEEpIC esophageal epithelial cell line (shown as a peak; ENCODE/UW) and KEJ293 cells form ENCODE with Factor Motifs. RNA levels = Messenger RNA (mRNA) expression from this region in esophagus, gastric and peripheral blood mononuclear (PBM) cells, as well as small RNA sequencing (smRNA-Seq ) detection of noncoding RNAs in peripheral blood mononuclear cells (PBMC). *Track sources and acknowledgements for the UCSC genome, ENCODE, The NIH ROADMAP databases and extracted tracks* [*http://genome.ucsc.edu/goldenPath/credits.html#human_credits*](http://genome.ucsc.edu/goldenPath/credits.html)

**Supplementary Figure 2.** Genome Browser (http://genome.ucsc.edu/) image of 19p13.12 genomic region and genes on human assembly hg19 based on NIH Epigenomics Roadmap and ENCODE data. Coding & noncoding genes= Gencode Version 19 showing long intergenic long noncoding RNAs (*AC004791.2*, *UCA1* and *AC004510.2*) and p450 protein coding genes (*CYP4F2* and *CYP4F11*). CpG islands: Green bars. Regulatory regions= chromatin states from a hidden Markov Model [ChromHMM)] and core histone marks with H3K27 acetylation mark from normal blood cells [CD19 B cells and CD4 and CD8 T cells), esophagus, stomach mucosa (SM), gastric and stomach smooth muscle (SSM). Color key: Crimson, flanking promoter region; Hot Red, active promoter; Yellow: poised enhancer; Dark gray, repressed polycomb region; and Light gray, weak repressed polycomb region; Green, transcription. SNPs= For clarity only the location of rs12461816 is shown. Methylation= Bisulphite-sequencing of gastric and esophageal tissues (Roadmap). DNase clusters= Open chromatin (DNA) structure in 125 ENCODE cells lines. TF/protein binding=Chip-seq DNA and protein interactions for transcription factors and protein(s) in 161 ENCODE cell lines. RNA levels = Messenger RNA (mRNA) expression from this region in esophagus, gastric and peripheral blood mononuclear (PBM) cells, as well as small RNA sequencing (smRNA-Seq ) detection of noncoding RNAs in peripheral blood mononuclear cells (PBMC). *Track sources and acknowledgements for the UCSC genome, ENCODE, The NIH ROADMAP databases and extracted tracks* [*http://genome.ucsc.edu/goldenPath/credits.html#human_credits*](http://genome.ucsc.edu/goldenPath/credits.html)

**Supplementary Table 1. Top SNPs in the NCI and Henan GWAS associated with family history of UGI cancer in ESCC cases^a^**

| **Gene** | **SNP (major, minor)** | **Chr.** | **NCI GWAS** | | | | | |  | **Henan GWAS** | | | | | |
| --- | --- | --- | --- | --- | --- | --- | --- | --- | --- | --- | --- | --- | --- | --- | --- |
|  |  |  | **n1** | **n2** | **A1** | **A2** | **OR ^b^** | ***P* ^b^** |  | **n1** | **n2** | **A1** | **A2** | **OR ^b^** | ***P* ^b^** |
| *GRIK4* | rs140792366 (C, G) | 11q23.3 | 540 | 1398 | 0.026 | 0.015 | 3.91 | 0.0006 |  | 492 | 868 | 0.028 | 0.014 | 4.62 | 0.0003 |
| *BC047542* | rs117453803 (A, T) | 2q37.1 | 540 | 1398 | 0.074 | 0.051 | 2.32 | 7.2×10^-5^ |  | 492 | 868 | 0.076 | 0.053 | 1.85 | 0.003 |
|  | rs187481103 (A, C) | 17p13.2 | 540 | 1399 | 0.026 | 0.013 | 5.80 | 1.56×10^-5^ |  | 492 | 868 | 0.022 | 0.013 | 2.84 | 0.01 |
| *COLA1L, COL21A1* | rs9357885 (A, T) | 6p12.1 | 540 | 1398 | 0.183 | 0.137 | 1.64 | 1.86×10^-5^ |  | 492 | 868 | 0.175 | 0.143 | 1.35 | 0.01 |
|  | rs2049728 (T, C) | 2p24.1 | 540 | 1399 | 0.251 | 0.310 | 0.77 | 0.0007 |  | 492 | 869 | 0.259 | 0.309 | 0.76 | 0.002 |
|  | rs57921607 (A, C) | 2q32.1 | 540 | 1398 | 0.163 | 0.134 | 1.52 | 0.002 |  | 492 | 868 | 0.163 | 0.127 | 1.71 | 0.0005 |
| *CYP19A1* | rs186503151 (C, T) | 15q21.2 | 541 | 1399 | 0.028 | 0.014 | 5.81 | 1.86×10^-5^ |  | 492 | 869 | 0.024 | 0.013 | 2.41 | 0.02 |
|  | rs2372415 (C, G) | 7q35 | 540 | 1398 | 0.285 | 0.228 | 1.39 | 0.0002 |  | 492 | 868 | 0.278 | 0.233 | 1.30 | 0.007 |
|  | rs12631160 (T, C) | 3p22.3 | 540 | 1398 | 0.046 | 0.026 | 2.18 | 0.0005 |  | 493 | 869 | 0.048 | 0.028 | 2.00 | 0.004 |
| *DLGAP1* | rs79747906 (T, C) | 18p11.31 | 540 | 1398 | 0.068 | 0.051 | 1.70 | 0.007 |  | 492 | 868 | 0.081 | 0.053 | 2.16 | 0.0002 |
| *STK31* | rs1549391 (C, T) | 7p15.3 | 541 | 1398 | 0.043 | 0.071 | 0.59 | 0.0006 |  | 492 | 869 | 0.037 | 0.066 | 0.59 | 0.004 |
| *NXPH1, hCG_2009575* | rs2285970 (T, C) | 7p21.3 | 540 | 1398 | 0.034 | 0.020 | 1.90 | 0.02 |  | 492 | 868 | 0.040 | 0.017 | 3.20 | 5.21×10^-5^ |
|  | rs1073209 (T, C) | 3p22.3 | 540 | 1399 | 0.089 | 0.069 | 1.43 | 0.01 |  | 493 | 869 | 0.101 | 0.063 | 1.85 | 7.55×10^-5^ |
| *C12orf5, FGF23* | rs1046165 (T, C) | 12p13.32 | 541 | 1399 | 0.519 | 0.457 | 1.28 | 0.0007 |  | 493 | 869 | 0.540 | 0.478 | 1.28 | 0.003 |
|  | rs59635584 (G, T) | 20q13.31 | 540 | 1398 | 0.033 | 0.020 | 2.39 | 0.003 |  | 492 | 868 | 0.038 | 0.020 | 2.70 | 0.0007 |
| *ATP1B2, TP53, p53* | rs1050533 (T, C) | 17p13.1 | 540 | 1398 | 0.522 | 0.463 | 1.33 | 0.0002 |  | 492 | 868 | 0.494 | 0.447 | 1.25 | 0.01 |
| *UCA1* | rs12461816 (C, T) | 19p13.12 | 540 | 1398 | 0.205 | 0.177 | 1.25 | 0.03 |  | 492 | 868 | 0.233 | 0.173 | 1.58 | 2.96×10^-5^ |
| *COLA1L, COL21A1* | rs2745751 (A, C) | 6p12.1 | 540 | 1398 | 0.105 | 0.083 | 1.39 | 0.02 |  | 493 | 869 | 0.121 | 0.075 | 1.69 | 0.0001 |
| *MPDU1, FXR2, SHBG* | rs58614441 (T, C) | 17p13.1 | 540 | 1398 | 0.187 | 0.149 | 1.44 | 0.0009 |  | 492 | 868 | 0.185 | 0.147 | 1.43 | 0.003 |

^a^SNPs are ordered based on the *P*-values in the meta-analysis, same as that in Table 2. n1 is the number of cases with family history while n2 is the number of cases without family history. A1 is the allele frequency of the minor allele (effect allele) in cases with family history and A2 is the allele frequency of the minor allele (effect allele) in cases without family history.

^b^The *P*-values and ORs for the SNP (per one minor allele) were calculated from unconditional logistic regression models using genotype-trend tests adjusted for age, sex and sub-study (for the analysis of NCI study, which includes NCI Shanxi and NIT).

**Supplementary Table 2. The associations for the top meta-GWAS SNPs in the Henan replication: the secondary model ^a^**

| **Gene** | **Chr.** | **Original SNP in the discovery phase(major, minor)** | **Henan replication (second model)** | | | | | | |
| --- | --- | --- | --- | --- | --- | --- | --- | --- | --- |
|  |  |  | **SNP (major, minor)** | **n1** | **n2** | **A1** | **A2** | **OR ^b^** | ***P* ^b^** |
| *GRIK4* | 11q23.3 | rs140792366 (C, G) | rs140792366 (C, G) | 1937 | 3135 | 0.007 | 0.005 | N/A^f^ | N/A^f^ |
| *BC047542* | 2q37.1 | rs117453803 (A, T) | rs73997003 (G, A)^c^ | 1937 | 3136 | 0.076 | 0.067 | 1.13 | 0.13 |
| *WSCD1,KIAA0523* | 17p13.2 | rs187481103 (A, C) | rs184911713 (G)^d^ | 1937 | 3135 | 0 | 0 | N/A^f^ | N/A^f^ |
| *COLA1L,COL21A1* | 6p12.1 | rs9357885 (A, T) | rs6459122 (C, T)^c^ | 1935 | 3135 | 0.093 | 0.094 | 0.98 | 0.76 |
|  | 2p24.1 | rs2049728 (T, C) | rs2049728 (T, C) | 1935 | 3135 | 0.288 | 0.296 | 0.96 | 0.37 |
|  | 2q32.1 | rs57921607 (A, C) | N/A^e^ | N/A^e^ | N/A^e^ | N/A^e^ | N/A^e^ | N/A^e^ | N/A^e^ |
| *CYP19A1* | 15q21.2 | rs186503151 (C, T) | rs141703242 (C) ^d^ | 1937 | 3136 | 0 | 0 | N/A^f^ | N/A^f^ |
|  | 7q35 | rs2372415 (C, G) | rs2372415 (C, G) | 1935 | 3135 | 0.257 | 0.258 | 1.00 | 0.94 |
|  | 3p22.3 | rs12631160 (T, C) | rs12631160 (T, C) | 1935 | 3135 | 0.044 | 0.047 | 0.95 | 0.63 |
| *DLGAP1* | 18p11.31 | rs79747906 (T, C) | rs79747906 (T, C) | 1937 | 3136 | 0.076 | 0.062 | **1.24** | **0.008** |
| *STK31* | 7p15.3 | rs1549391 (C, T) | rs1549391 (C, T) | 1937 | 3136 | 0.053 | 0.051 | 1.06 | 0.54 |
| *NXPH1,hCG_2009575* | 7p21.3 | rs2285970 (T, C) | rs2285970 (T, C) | 1935 | 3135 | 0.031 | 0.036 | 0.84 | 0.13 |
|  | 3p22.3 | rs1073209 (T, C) | rs4955227 (T, G)^c^ | 1937 | 3136 | 0.072 | 0.071 | 1.02 | 0.81 |
| *C12orf5,FGF23* | 12p13.32 | rs1046165 (T, C) | rs1046165 (T, C) | 1937 | 3136 | 0.476 | 0.479 | 0.99 | 0.77 |
|  | 20q13.31 | rs59635584 (G, T) | rs59635584 (G, T) | 1937 | 3136 | 0.020 | 0.020 | 0.98 | 0.88 |
| *ATP1B2,TP53,p53* | 17p13.1 | rs1050533 (T, C) | rs1050541 (T, G)^c^ | 1937 | 3135 | 0.418 | 0.410 | 1.03 | 0.52 |
| *UCA1* | 19p13.12 | rs12461816 (C, T) | rs12461816 (C, T) | 1937 | 3136 | 0.217 | 0.202 | **1.09** | **0.08** |
| *COLA1L,COL21A1* | 6p12.1 | rs2745751 (A, C) | rs1883703 (A, T)^c^ | 1935 | 3135 | 0.079 | 0.082 | 0.95 | 0.53 |
| *MPDU1*,*FXR2*,*SHBG* | 17p13.1 | rs58614441 (T, C) | rs34416693 (G, A)^c^ | 1936 | 3135 | 0.232 | 0.230 | 1.00 | 0.98 |

^a^ SNPs are ordered based on the *P*-values in the meta-analysis (same as that in Table 2). n1 is the number of cases with family history while n2 is the number of cases without family history. A1 is the allele frequency of the minor allele (effect allele) in cases with family history and A2 is the allele frequency of the minor allele (effect allele) in cases without family history. ESCC cases with family history were defined as those that have one or more relative(s) within first-degree relatives diagnosed with UGI cancer.

^b^The *P*-values and ORs for the SNP (per one minor allele) were calculated from unconditional logistic regression models using genotype-trend tests adjusted for age, sex and sub-study (for the analysis of NCI study, which includes NCI Shanxi and NIT).

^c^ Surrogate SNPs were selected to replace the original SNP from the meta-analysis of the NCI and Henan GWAS. The surrogates were located within 200kb on either side of each targeted SNP and were selected based on the r^2^ with the targeted SNP in the genotype data from 1000 Genomes project JPT+CHB population. The linkage r^2^ was 1.00 for rs73997003 with rs117453803, 0.62 for rs6459122 with rs9357885, 0.93 for rs4955227 with rs1073209, 0.58 for rs1050541 with rs1050533, 0.94 for rs1883703 with rs2745751, and 0.59 for rs34416693 with rs58614441.

^d^For rs187481103 and rs186503151, we were unable to find good surrogates that could survive the assay design. Given the monoallelic nature of the tested surrogates (rs184911713 and rs141703242) in our study population, we were essentially unable to test/replicate the findings for these two SNPs.

^e^ This SNP (rs57921607) and its surrogate(s) failed in the follow-up study.

^f^ ORs and P values not calculated because of the extremely rare minor allele.

**Supplementary Table 3. All suggestive associations with P<0.05 from the eQTL analysis of rs79747906 and rs12461816^a^**

| **SNP** | **rs79747906 (risk allele C, 18p11.31)^b^** | **rs12461816 (risk allele T, 19p13.12)** |
| --- | --- | --- |
| **Gene Annotation** | *DLGAP1,* intergenic | *UCA1*, intergenic |
| **eQTL in esophagus** | *RP11-838N2.5* (β= 0.55, P=0.013) Muscularis *RP13-270P17.2* (β= -0.36, P=0.018) GEJ *RP13-270P17.3* (β=-0.35, P=0.032) GEJ | ***AC004791.2* (β= 0.60, P=6.00×10^-7^) Muscularis *CYP4F24P* (β= 0.66, P=4.50×10^-4^) GEJ *AC004791.2* (β= 0.37, P=7.30×10^-4^) Mucosa *CYP4F11* (β= -0.37, P=8.50×10^-4^) Mucosa** *AC004510.3* (β= -0.23, P=1.00×10^-3^) Mucosa AC005336.4 (β= -0.40, P=1.20×10^-3^) Muscularis *CYP4F2* (β= -0.33, P=1.40×10^-3^) Mucosa  *AC005336.4* (β= -0.34, P=3.10×10^-3^) Mucosa *SYDE1* (β= 0.31, P=3.30×10^-3^) GEJ  *LLNLR-249E10.1* (β= 0.50, P=3.80×10^-3^) GEJ *CYP4F11* (β= -0.35, P=4.80×10^-3^) Muscularis  *CHERP* (β= -0.11, P=9.10×10^-3^) Mucosa *CTC-429P9.3* (β= 0.14, P=0.010) Muscularis *CYP4F24P* (β= 0.31, P=0.012) Muscularis *CYP4F24P* (β= 0.29, P=0.014) Mucosa  *NWD1* (β= -0.19, P=0.015) Muscularis *UCA1* (β=-0.12, P=0.021) Mucosa  *UCA1* (β= -0.12, P=0.021) Mucosa *CTC-429P9.5* (β= 0.18, P=0.024) Muscularis  *CTD-3222D19.5* (β= 0.28, P=0.026) Mucosa *CTD-2562J15.4* (β= -0.40, P=0.039) GEJ  *SYDE1* (β= 0.09, P=0.042) Muscularis *TMEM38A* (β= -0.25, P=0.047) GEJ |
| **eQTL in stomach** | *RP13-270P17.3* (β=0.58, P= 8.30×10^-3^)  *RP13-270P17.2* (β= 0.47, P=0.021) | ***AC004791.2* (β= 0.67, P=9.50×10^-7^)**  *CTC-429P9.2* (β= 0.21, P=0.020)  *CYP4F12* (β= 0.15, P=0.027)  *CYP4F2* (β= -0.23, P=0.032)  *CTC-429P9.5* (β= 0.19, P=0.039)  *LLNLR-246C6.1* (β= 0.28, P=0.041)  *CTD-2231E14.8* (β= 0.14, P=0.048) |
| **eQTL in blood** | *LPIN2* (β= 0.26, P= 6.70×10^-3^) | ***AC004791.2* (β= 0.38, P=2.5×10^-5^)**  *CYP4F12* (β=0.18, P=0.013)  *CASP14* (β=-0.22, P=0.016) |
| **No. of eQTL tests (significance *P*)** | 17 tests (*P*<2.94×10^-3^) | 57 tests (*P*<8.77×10^-4^) |

^a^Expression quantitative trait loci (eQTL) analysis for the risk allele were conducted in normal esophageal tissues: esophageal mucosa[Mucosa]; esophageal muscularis [Muscularis]; and gastroesophageal junction [GJE], normal stomach mucosa, and the whole blood. We examined coding and non-coding genes *in cis* or located within a 1MB of the signal and known to be expressed at the mRNA in the target tissue. Results were obtained from the Genotype-Tissue Expression (GTEx) Project (<http://www.gtexportal.org/home/>). *P-*values were calculated based on linear regression between log and quantile normalized RNA-seq expression values and imputation-based genotype with 3 genotyping principal components, 15 peer factor, and gender as covariates. All suggestive associations with P<0.05 are shown here. The highlighted associations are also shown in Table 3.

^b^No significant association after considering multiple comparisons for number of tests.

**Supplementary Table 4. Information on genes with suggestive associations in the eQTL analysis in Table 3 and SI Table 3**

| **Ensembl Gene ID** | **Band** | **Gene Name** | **Gene Type** |
| --- | --- | --- | --- |
| ENSG00000267308 | 19p13.12 | *AC004510.3* | lincRNA |
| ENSG00000267453 | 19p13.12 | *AC004791.2 ^a^* | lincRNA |
| ENSG00000267056 | 19p13.12 | *AC005336.4* | processed_pseudogene |
| ENSG00000105141 | 19p13.12 | *CASP14* | protein_coding |
| ENSG00000085872 | 19p13.11 | *CHERP* | protein_coding |
| ENSG00000268087 | 19p13.11 | *CTC-429P9.2* | lincRNA |
| ENSG00000269044 | 19p13.11 | *CTC-429P9.3* | sense_intronic |
| ENSG00000267904 | 19p13.11 | *CTC-429P9.5* | sense_intronic |
| ENSG00000269243 | 19p13.11 | *CTD-2231E14.8* | antisense |
| ENSG00000267033 | 19p13.11 | *CTD-2562J15.4* | antisense |
| ENSG00000269578 | 19p13.11 | *CTD-3222D19.5* | sense_intronic |
| ENSG00000171903 | 19p13.12 | *CYP4F11 ^a^* | protein_coding |
| ENSG00000186204 | 19p13.12 | *CYP4F12* | protein_coding |
| ENSG00000186115 | 19p13.12 | *CYP4F2* | protein_coding |
| ENSG00000267594 | 19p13.12 | *CYP4F24P ^a^* | unprocessed_pseudogene |
| ENSG00000268673 | 19p13.12 | *LLNLR-246C6.1* | lincRNA |
| ENSG00000268673 | 19p13.12 | *LLNLR-249E10.1* | lincRNA |
| ENSG00000101577 | 18p11.31 | *LPIN2* | protein_coding |
| ENSG00000188039 | 19p13.11 | *NWD1* | protein_coding |
| ENSG00000266578 | 18p11.31 | *RP11-838N2.5* | lincRNA |
| ENSG00000265399 | 18p11.31 | *RP13-270P17.2* | antisense |
| ENSG00000272688 | 18p11.31 | *RP13-270P17.3* | lincRNA |
| ENSG00000105137 | 19p13.12 | *SYDE1* | protein_coding |
| ENSG00000072954 | 19p13.11 | *TMEM38A* | protein_coding |
| ENSG00000273782 | 19p13.12 | *UCA1* | misc_RNA |

^a^ With significant *P* values after multiple comparison adjustment in Table 3.
