# Supplementary material for: GWAS follow-up study of esophageal squamous cell carcinoma identifies potential genetic loci associated with family history of upper gastrointestinal cancer
Source: Sci Rep. 2017 Jul 5;7:4642. doi: 10.1038/s41598-017-04822-2 (PMC5498542; doi:10.1038/s41598-017-04822-2)

Supplementary Figure 1

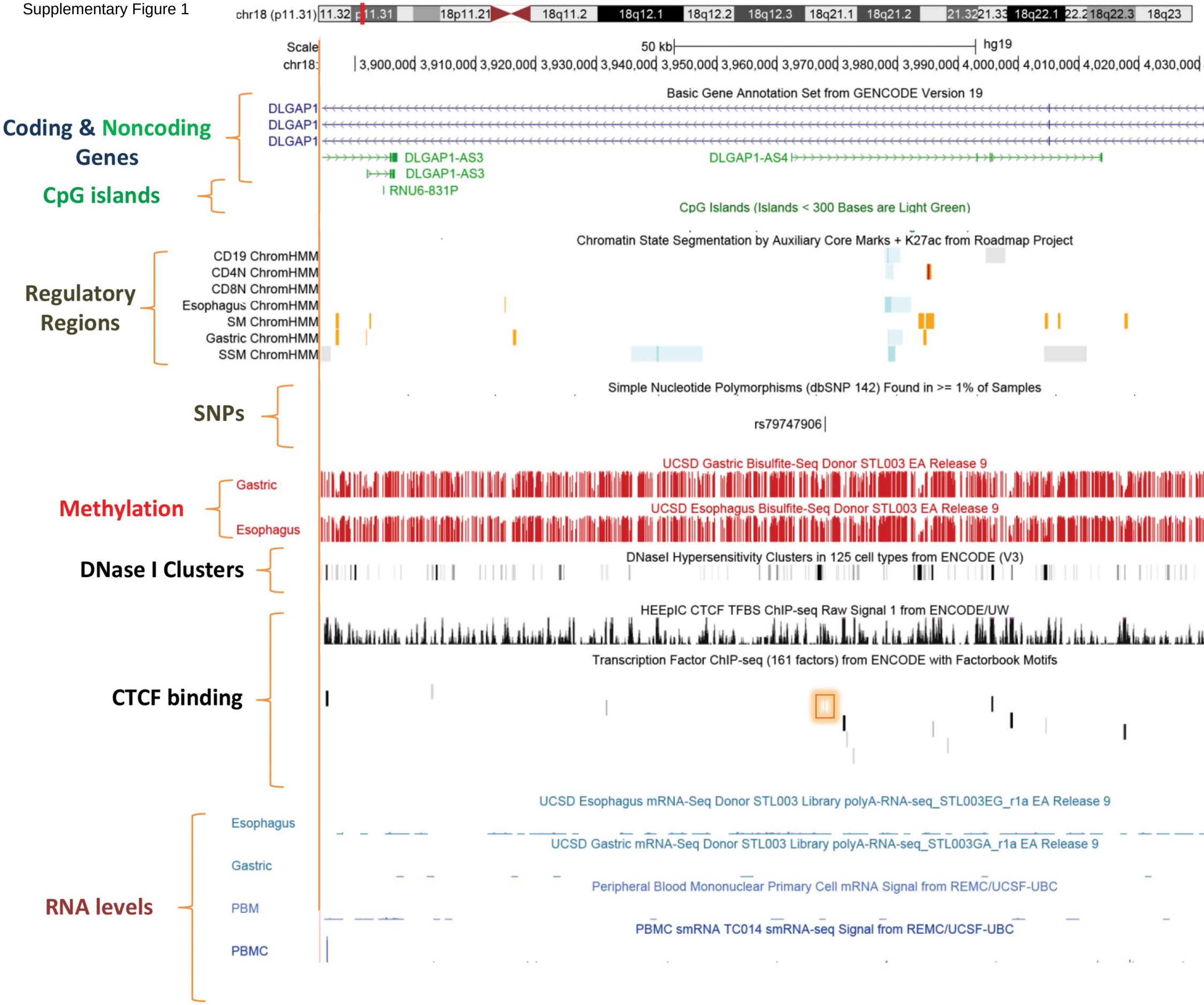

Supplement: Supplementary file 2 — Supplementary information [file 41598_2017_4822_MOESM2_ESM.pdf]
